# The Relationship Between Physical Activity, Sleep, and Hallucinations in Adults with Charles Bonnet Syndrome

**DOI:** 10.3390/vision10030040

**Published:** 2026-07-05

**Authors:** Jarrod Hollis, Rohit Narayan, Eldre W. Beukes, Rosie Lindsay, Aliyah Bharwani, Umair Mughal, Ikra Yaqoob, Justin A. Haegele, Scot Muirden, Peter M. Allen

**Affiliations:** 1 Vision and Hearing Sciences Research Centre, Anglia Ruskin University, Cambridge CB1 1PT, UK; jarrod.hollis1@nhs.net (J.H.); rohit.narayan@aru.ac.uk (R.N.); eldre.beukes@aru.ac.uk (E.W.B.); aliyahbharwani21@gmail.com (A.B.); umairm82@gmail.com (U.M.); yaqoobikra8@gmail.com (I.Y.); 2 THIS Institute (The Healthcare Improvement Studies Institute), Department of Public Health and Primary Care, University of Cambridge, Cambridge CB1 8RN, UK; rosie.lindsay@thisinstitute.cam.ac.uk; 3 Center for Movement, Health & Disability, Department of Human Movement Studies & Special Education, Old Dominion University, Norfolk, VA 23529, USA; jhaegele@odu.edu; 4 Charles Bonnet Syndrome Foundation, Melbourne, 247-251 Flinders Lane, Melbourne, VIC 3000, Australia; scot@charlesbonnetsyndrome.org

**Keywords:** Charles Bonnet Syndrome, hallucinations, physical activity, sleep, vision impairment

## Abstract

Physical activity levels, hallucination experiences, sleep quality, and perceived stress were assessed using a survey administered to 57 adults with a confirmed diagnosis of Charles Bonnet Syndrome (CBS). Physical activity demonstrated a significant negative correlation with hallucination severity (r = –0.335, *p* = 0.011, two-tailed). A significant negative correlation was also observed between physical activity and perceived stress (r = –0.497, *p* < 0.001, two-tailed). In contrast, no significant association was identified between hallucination frequency and sleep disruption or insomnia. These findings suggest that engaging in appropriate levels of physical activity may help reduce the severity of hallucinations and mitigate stress among individuals with CBS. The observation that higher levels of physical activity are associated with reduced hallucination burden may help the development of evidence-based management strategies for CBS. Additionally, the finding that physical activity was significantly related to hallucination severity, whereas sleep disruption was not, merits deeper exploration in future research.

## 1. Introduction

Charles Bonnet Syndrome (CBS) is a condition linked to sight loss in which people experience visual hallucinations despite the absence of real visual input [1]. It affects roughly one in six individuals with vision impairment [2], amounting to an estimated 47.2 million people worldwide [2,3]. These hallucinations can often cause fear or distress [4]. Although CBS occurs across a range of eye diseases, no strong associations have been found with any specific ocular condition, gender, or social factor [5]. There is also no universally accepted threshold for how much vision must be lost before hallucinations occur, though two commonly suggested cut-offs for visual acuity in the better-seeing eye are 0.3 LogMAR (6/12 or 20/40) and 0.4 LogMAR (6/15 or 20/50) [6].

Individuals with CBS report a diverse range of hallucinatory experiences, which are typically classified into *simple* and *complex* forms of visual perceptual disturbance [7]. Simple hallucinations consist of elementary visual phenomena such as geometric patterns, lines, grids, brickwork configurations, lights, or colours. In contrast, complex hallucinations involve more elaborate and structured imagery, including recognisable objects, architectural forms, people, landscapes, or fully developed scenes [4]. These experiences are widely theorised to arise from cortical mechanisms that compensate for diminished afferent visual input by generating internally constructed imagery [7]. The factors determining why some individuals experience only simple hallucinations while others perceive more intricate visual content remain unclear [4,7]. Crucially, the hallucinations associated with CBS are not attributable to psychiatric pathology [8,9]. A diagnosis is generally established when visual hallucinations occur in the context of significant vision loss and in the absence of comorbid neuropsychiatric disorders, such as schizophrenia [8,9].

Awareness of CBS remains limited, and this lack of understanding contributes to the stigma frequently associated with the experience of visual hallucinations [8]. These hallucinations are often mistakenly attributed to underlying mental health disorders [10]. However, the phenomenology of hallucinations in CBS differs markedly from that observed in psychotic conditions, as individuals with CBS typically retain full insight and recognise that the visual phenomena are not real. Indeed, loss of insight or the presence of cognitive impairment is used clinically to differentiate CBS from neuropsychiatric conditions such as dementia [10]. Despite this, hallucinations can be distressing and may disrupt activities of daily living [11]. Concerns about being mislabelled or stigmatised are key reasons why many individuals experiencing CBS do not disclose their symptoms or seek a formal diagnosis [10,11].

This study investigated the relationship between the experience of hallucinations, physical activity, sleep, and perceived stress. The aim was to determine whether any of these factors could potentially mediate the severity of hallucinations that people experience.

## 2. Materials and Methods

### 2.1. Study Design

A cross-sectional survey study design was used to explore experiences of hallucinations and physical activity for individuals with CBS. Ethical approval was granted by the Faculty of Psychology and Sport Sciences at Anglia Ruskin University, Cambridge, UK (ETH2223-120). The Equator network checklist for reporting internet surveys was used as a guide for reporting the methods and results.

### 2.2. Survey Development

Items for the survey were identified through an iterative process focusing on clinical experience. A list of possible questions was drafted, modified, and trialled, with the help of three people with CBS, to produce the final survey (see Supplementary Material S1). The questionnaire was administered via Qualtrics and consisted of

- Demographic information such as age, gender, geographical location, ethnicity (4 questions).
- Vision impairment characteristics (3 questions).
- CBS-related information, such as diagnosis, visual phenomena experiences (2 questions).
- Visual phenomena-related questions, including an estimate of the type (simple/complex), frequency, duration, association with lighting conditions, circumstances at the onset of the visual phenomena, what interventions reduce the visual phenomena, severity/impact of hallucinations, and who they have told about these visual phenomena. (10 questions).
- Perceived stress was assessed using a rating scale of 1–4, indicating no perceived stress (1), mild stress (2), moderate stress (3), and severe stress (4). These ratings were weighted according to the reported impact rating to generate a composite stress score, which was categorised for analysis into mild, moderate, and severe stress.

Physical activity information included both the amount and type of activity undertaken. The questions were based on the Physical Activity Scale for the Elderly (PASE), a standardised and widely used inventory for assessing physical activity levels [12,13,14,15]. The PASE evaluates activity performed during the preceding seven days across three domains: leisure-time physical activity, household and domestic tasks, and occupational or voluntary work. Leisure-time activities include walking, light sport, moderate sport, and strenuous physical activity. Participants reported the frequency of each activity using categorical response options (never; seldom, 1–2 days; sometimes, 3–4 days; often, 5–7 days), followed by the average duration per day where applicable. Household activities (e.g., light housework, heavy housework, home repairs, gardening, and caregiving) were recorded using yes/no responses. Occupational or voluntary activity was quantified based on the number of hours worked per week; weekly hours were divided by seven to estimate daily engagement. Participants also indicated the physical nature of their work, as only activities involving at least light physical exertion contribute to the final PASE score.

Each activity frequency was multiplied by empirically derived weightings that reflect relative energy expenditure. Weighted scores across all three domains were then summed to produce a total PASE score. Total scores typically range from 0 to over 400, with higher scores indicating greater levels of physical activity. The PASE inventory is considered a robust and effective measure of physical activity [14,15], and PASE scores have been shown to correlate well with other indicators of physical health and physical performance.

Sleep and fatigue have been reported as exacerbating factors in CBS [16]. However, sleep has not been systematically quantified in this population using validated assessment techniques. In the present study, information on sleep quality and associated symptoms was collected using the Insomnia Severity Index (ISI), a validated instrument designed to assess the nature, severity, and functional impact of insomnia symptoms [17]. The ISI demonstrates strong reliability and sensitivity in adult populations [18].

The ISI consists of seven items referring to the preceding two weeks and assesses difficulties with sleep onset, maintaining sleep, and early morning awakenings, as well as satisfaction with current sleep patterns, perceived impairment in quality of life, distress related to sleep problems, and interference with daily functioning. Each item is rated on a 5-point Likert scale (0–4), and scores are summed to produce a total score ranging from 0 to 28, with higher scores indicating greater insomnia severity. Total scores are typically categorised as follows: 0–7 = no clinically significant insomnia; 8–14 = subthreshold insomnia; 15–21 = moderate insomnia; and 22–28 = severe insomnia [17]. For the purposes of analysis in the present study, the first two categories were combined to create three groups to simplify interpretation: mild/no insomnia (0–14), moderate insomnia (15–21), and severe insomnia (22–28). Items for the survey were presented in a fixed sequential order—respondents were unable to change their responses once submitted. No identifiable data were collected.

### 2.3. Survey Distribution

Recruitment targeted individuals with CBS in the UK and the USA. Those residing in the United Kingdom were recruited through patient organisations and charities that support people with visual impairment, including Esme’s Umbrella, the Royal National Institute of Blind People (RNIB), the Macular Society, and Retina UK. In the United States, recruitment was facilitated through a listserv comprising adults with blindness or visual impairment who had expressed interest in participating in research. The survey was launched in December 2024 and remained open for a period of 16 weeks. Emails outlining the study purpose, inclusion criteria, estimated time commitment, and the anonymous and confidential nature of the survey were distributed twice during the 16-week data-collection period.

Eligibility was further assessed by asking participants whether they experienced hallucinations and whether they had received a formal diagnosis of CBS from a healthcare professional. There were no other exclusion criteria beyond a minimum age requirement of 18 years. Participants were required to provide online informed consent prior to beginning the survey, and the survey platform restricted responses to one submission per IP address.

### 2.4. Data Analysis

Data cleaning was initially undertaken. Statistical analysis was conducted using Excel and JASP 0.1.7.2.0. Descriptive statistics were calculated to include frequencies, medians, means, and standard deviations.

A weighted activity score based on the PASE system was calculated to take into account all activities ranging from light housework, strenuous activity, walking, volunteering, and other physical activity. Activity scores were calculated as a total derived across all of the subscales on the PASE. These scores ranged between 0 and 388 with a mean of 91.2, SD 76, and a median of 70.

Hallucinations were rated for frequency, duration, severity, and impact and given a weighted score across the totals (frequency × duration × severity). Hallucination scores ranged from 0.5 to 336 with a mean of 76, SD 90, and median of 42.

The sleep quality scores were formed using a simple scale as a weighted score derived from questions that probed difficulty in falling asleep, difficulty staying asleep, waking too early, and dissatisfaction with sleep. These scores ranged between 0 and 25, with a median of 10, and a mean of 11.0 (SD 6.3). The responses were also categorised in terms of reported sleep disturbance (mild, moderate, or severe).

The perceived stress ratings ranged between 0 and 4, with zero referring to experiencing no stress. The ratings were weighted using the stress impact rating (stress rating × stress impact) to produce a stress score and then categorised as mild, moderate, or severe.

## 3. Results

### 3.1. Characteristics of the Participants

Respondents were excluded from analysis if they did not fully complete the inventory. Data from 57 respondents were subjected to analysis. The mean age was 66 years (SD 14; range: 34 to 97 years), with 56% of the sample being over the age of 65 years. There was a higher proportion of female participants (37 females, 20 males). All the respondents reported ethnicity as White and were based either in the UK or the USA. Of the respondents, 75% had been formally registered as sight-impaired or severely sight-impaired. All participants had reported experiencing visual phenomena consistent with a diagnosis of Charles Bonnet Syndrome. Some of the participants who were not registered as sight-impaired were awaiting formal diagnosis. Only three participants (6% of the respondents) stated that they had tried any type of strategy or intervention to help cope with the hallucinations. The strategies that were reported consisted of physical actions such as blinking a lot, changing the lighting in the room, or directing attention to something else, via speech or other sound.

#### 3.1.1. Experience of Hallucinations

The hallucinations experienced varied in type, frequency, and duration. A combination of simple and complex visual hallucinations was reported. Participants reported complex hallucinations (images of objects and visual scenes that included people and animals, for example, burning trees), which included simple hallucinations (geometric patterns, lights). There were no reports of simple hallucinations alone.

Hallucinations occurred for varying lengths of time, ranging from brief durations of a few seconds to many hours, and in some cases even continuously (31%). Respondents were not always aware of the triggers to these episodes.

Not all the participants reported that the hallucinations they experienced caused distress. A small number of respondents stated that there was no perceived stress related to their visual experiences (17%), whilst a larger proportion (83%) reported distress to some degree, which negatively affected their life and wellbeing.

Contrary to previous research, there was a significantly higher rate of hallucinations reported by male respondents, indicated by a significant effect of gender, *F* (1,55) = 7.35, *p* = 0.009, w^2^ = 0.099.

#### 3.1.2. Hallucinations and Levels of Insomnia

The mean sleep score was 11.09 (SD: 6.3, median 10, range 0–25). Sleep disturbance scores were also categorised as mild, moderate, or severe. The effect of insomnia on the levels of hallucination was analysed using ANOVA. Differences in hallucination levels for the categories of insomnia were not significant, *F* (2,54) < 1, *p* = 0.43.

### 3.2. Participation in Physical Activity

None of the respondents reported that they used physical activity as a strategy for coping with hallucinations; however, 22% reported that they had noticed a link between their activity levels and the experience of hallucinations. Only 27% of the sample attained the recommended level of physical activity (according to the norm for 65-year-olds on the PASE inventory), with only 13% reporting that they engaged in any strenuous activity.

Thirty-two percent of respondents reported sedentary or low levels of gentle activity, such as light housework. Many stated that their visual impairment was the reason for their lack of activity. Reasons for not doing physical activity included the hallucinations making physical activity more difficult (approx. 10%), difficulty in concentration and focus of attention (approx. 7%). General fatigue (approx. 10%) was also noted as a contributing factor. For people with visual impairment, mobility generally can be difficult, with vigorous activity being particularly problematic. A small number of respondents (17%) reported that they were aware that on days when they were more active, they seemed to suffer fewer or less concerning hallucinations; the majority of respondents remained unaware of any association.

**Figure 1:**
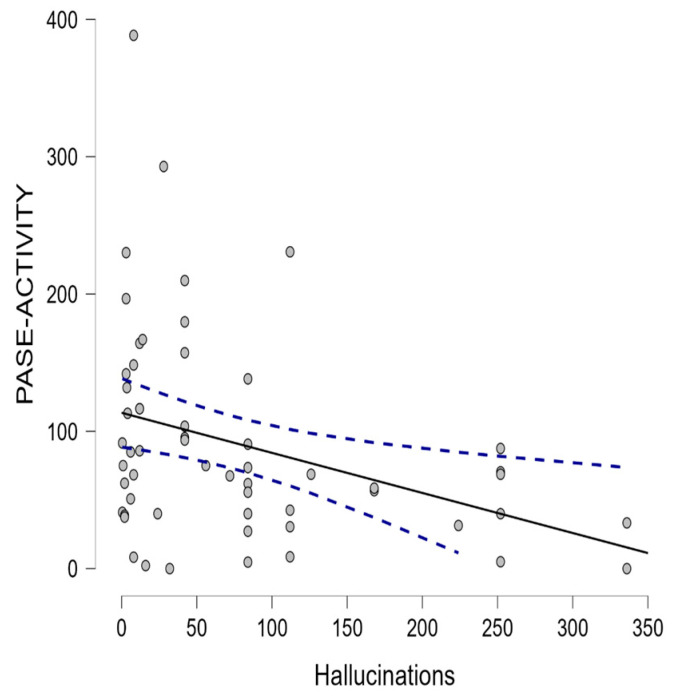
Relationship between hallucination frequency and PASE-activity. PASE activity scores and associated hallucination ratings are plotted, illustrating the line of best fit (solid line) and 95% confidence interval (dashed lines).

A correlational analysis was conducted using the hallucination ratings, the ratings for sleep quality, and the PASE ratings of physical activity. As seen in Figure 1, there was a significant negative correlation between total PASE physical activity and hallucinations [*Rho* = −0.335, *p* = 0.011, Fisher’s *Z* = −0.349, SE effect 0.138, two-tailed].

Direct relationships between hallucinations and sleep were not significant, whereas there were significant negative relationships between PASE activity and stress, and PASE activity and hallucinations (see Table 1).

**Figure 2:**
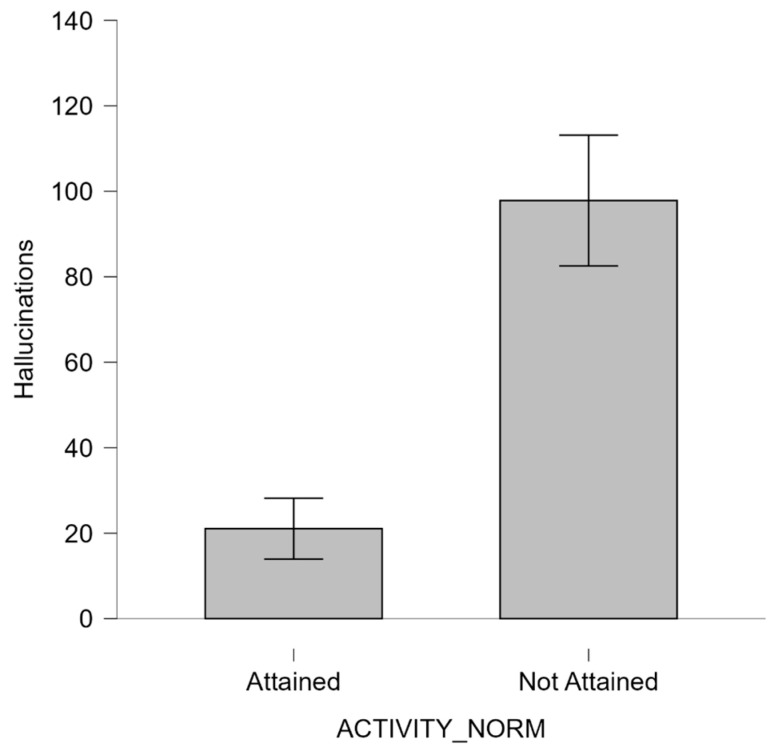
Level of hallucinations for respondents who attained or did not attain the average levels of physical activity according to the PASE study. Error bars show the SE.

Activity data were also categorised into two groups: those who attained or exceeded the normal expected PASE activity for age, and those who did not, taking the hallucination ratings as a dependent variable. These data were subjected to ANOVA. As seen in Figure 2, there was a significant decrease in levels of hallucination for people who attained the recommended levels of physical activity compared to those who did not *F* (1,55) = 9.4, *p* = 0.003 (effect size w^2^ = 0.129).

### 3.3. Physical Activity and Levels of Stress

**Figure 3:**
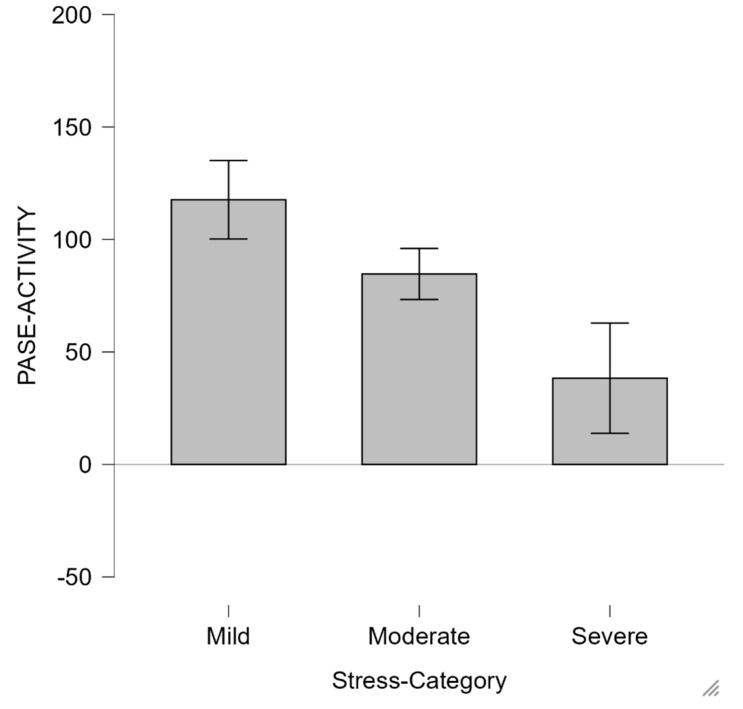
Level of PASE activity for respondents who were categorised as experiencing mild, moderate, or severe stress. Error bars illustrate SE.

Activity data were also considered with respect to the stress levels reported by the participants. Stress levels were categorised as mild, moderate, and severe. There was a significant trend with respondents who reported higher levels of physical activity, being categorised as experiencing lower levels of stress. *F* (2,54) = 4.12, *p* = 0.02 (effect size w^2^ = 0.09) (Figure 3). Post hoc analysis with contrasts of means indicated the differences occurred between the mild and severe categories (mean activity for mild stress 117, SD = 85, mean activity for severe stress 38, SD = 73; THSD mean difference 79.3, df = 54, *t* = 2.8, *p* = 0.09).

## 4. Discussion

Evidence-based strategies for reducing the negative impact of hallucinatory experiences remain limited. To advance understanding of potential management approaches, the present study examined the relationships among participants’ hallucination experiences, levels of physical activity, and perceived stress. The aim was to determine whether any of these variables might serve as potential mediators of hallucination severity. The findings are presented in the following section, alongside a discussion of study limitations and recommendations for future research.

Despite the relatively high prevalence of CBS, there remains no clear consensus regarding its diagnostic criteria. Research examining effective interventions to mitigate the impact of CBS on daily functioning is limited [19]. Current management for individuals with sight loss primarily centres on the use of low-vision aids. Pharmacological approaches, including the prescription of tranquillizers and antidepressants, are sometimes employed with the aim of reducing hallucinatory experiences, although evidence supporting their efficacy is inconsistent [19,20,21,22,23]. A small number of alternative management strategies have been proposed; however, the empirical foundation for these approaches remains weak [19].

There is a need to identify effective strategies for reducing both the frequency and the functional impact of hallucinations in individuals with CBS. One potential avenue for improving symptom management is to examine the factors associated with the onset and persistence of hallucinatory experiences. Psychosocial variables, including loneliness and social isolation, have been implicated in the occurrence of hallucinations [24,25]. Additional factors known to influence overall wellbeing and both physical and mental health—such as stress levels and sleep quality—may also play a contributory role.

Stress is known to impact wellbeing generally and is also a well-established contributing factor to physical and mental health. How different people experience and perceive stressful situations is highly individualistic [26]. It has long been established that perceived stress is as important as actual stress itself [27]. The cognitive-psychological factors of a particular individual may be the reason why some people react to stress in a more positive way than others [28]. Strategies that aim to reduce stress may, in turn, mitigate or reduce the severity of the hallucinations. Experiencing stress has links with the quality of sleep. Inadequate sleep is associated with a decline in both physical and mental health [29,30]. Higher levels of stress increase cortisol levels, making it harder to both fall asleep and stay asleep. Whilst the experience of good quality of sleep is well known to mediate stress [30], higher levels of stress are known to significantly disrupt sleep quality, thus creating a negative cycle which impacts wellbeing. This cycle of stress has been attributed to the hypothalamic–pituitary–adrenal [31]. Chronic lack of sleep over extended periods can also produce a range of altered perceptual experiences, which include hallucinations [32,33]. Both the perception of stress and quality of sleep are known associates of physical activity, with higher levels of physical activity being associated with reduced stress and improved health and psychological wellbeing [12,13,34]. Given that all these factors are associated with each other [35,36,37], changes to one of these modifiable factors may be one way of managing the experience of hallucinations. One such factor would be physical activity. The role of physical activity in the experiences of patients with CBS has received little attention in the past. Exploring the role of physical activity on the frequency of hallucinations in individuals with CBS could provide insights into further managing CBS.

### 4.1. Effect of Activity, Sleep, and Perceived Stress

Our correlational analysis revealed a significant negative association between physical activity levels and the severity of hallucinations reported by individuals with CBS. This finding was further supported by the ANOVA results, which indicated that participants who achieved the age-appropriate recommended level of physical activity experienced fewer hallucinations. Previous research has similarly shown that hallucinations tend to intensify during periods of heightened stress [6,9,36]. Consistent with this, our data demonstrated that individuals with higher levels of physical activity were more likely to report lower perceived stress. The significant relationship observed between stress and physical activity aligns with existing literature [27,28,32]. No significant association was identified between sleep and hallucination severity; however, this null finding may reflect limitations in the measures used to assess and quantify sleep experiences.

### 4.2. General Discussion

Vision impairment can affect nearly all domains of an individual’s life and has been consistently associated with poorer physical and mental health outcomes [27]. Consequently, interventions that reduce the risk of adverse physical health are essential for addressing health inequalities and the reduced life expectancy observed among people with sight loss [38,39,40]. Notably, substantial disparities exist in levels of physical activity engagement. Individuals with uncorrectable vision impairment are reported to be twice as likely to be physically inactive compared with those without sight loss [38].

Burton et al. [29] propose that facilitators and barriers to physical activity among people with sight loss operate across three domains: (1) psychological factors; (2) opportunity and access; and (3) societal and policy-level influences. These domains align closely with Crawford et al.’s [30] hierarchical model of leisure constraints, which conceptualises barriers to physical activity at three levels: intrapersonal, interpersonal, and structural (e.g., availability of facilities, environmental conditions, and resource provision). According to Crawford et al. [30], these constraints are hierarchical in nature, with intrapersonal barriers needing to be addressed before interpersonal and, subsequently, structural barriers can be effectively overcome.

These findings highlight the presence of multiple barriers to physical activity and underscore the need for tailored provision and specific guidance for individuals with CBS. The development of specialist physical activity groups that offer appropriate environments and trained support staff would likely benefit people with vision impairment more broadly. Such provision may enhance confidence, promote engagement, and encourage participation in activities within settings designed to accommodate their specific needs. However, the establishment and sustained resourcing of specialist programmes present significant challenges, particularly in relation to funding and long-term service provision [38].

Sociodemographic disparities in physical activity are also evident among people with sight loss. Previous research indicates that women with visual impairment may be less likely to engage in physical activity than men, and that individuals from Black and Asian ethnic backgrounds are more likely to be inactive compared with those identifying as white, white British, white other, or mixed ethnic groups [39]. However, in the present study, no respondents identified themselves as belonging to minoritised ethnic groups, limiting the extent to which these patterns could be examined within our sample.

Population-level research has demonstrated that lower household income and lower educational attainment are associated with an increased risk of physical inactivity [40]. When developing physical activity initiatives, it is therefore essential to identify the specific barriers faced by these population groups and to involve members of underrepresented communities—including community organisations and local leaders—in the planning and implementation of interventions designed to promote physical activity.

Psychological factors also contribute to the likelihood of inactivity. Individuals who experience a heightened fear of falling, low confidence, or reduced self-efficacy are particularly vulnerable to becoming inactive. It is therefore important that physical activity opportunities accommodate a wide range of abilities and include options that support gradual increases in low-intensity activity as an initial step toward improving overall activity levels. Enhancing the accessibility and safety of facilities and services is also critical for increasing individuals’ confidence that they can participate in physical activity safely and effectively.

### 4.3. Study Limitations and Further Research

Several limitations of this study should be considered when interpreting the findings. Despite the prevalence of CBS, identifying individuals with the condition proved challenging and hindered recruitment efforts. This difficulty was reflected in the absence of participants from ethnic minority groups, a pattern also noted in other health-related research [39]. In addition, the mean age of participants in the present sample (66 years) was somewhat younger than that reported in many previous studies. For example, Jan and Del Castillo [8] suggest that the mean age of individuals with CBS typically falls between 70 and 85 years. Variability in sampling across studies has been proposed as a potential explanation for inconsistencies in the literature regarding the signs and symptoms associated with CBS. This may also account for the higher levels of hallucinations reported by male participants in our sample, a finding that contrasts with earlier research. It has been argued that the severity of visual impairment, rather than gender, may be a more meaningful predictor of hallucinatory experience [41]. Seventy-five percent of participants in the present study were registered as sight-impaired, indicating that they had significant vision loss.

Data collection relied on self-report measures, which may introduce bias and inaccuracies due to the dependence on long-term memory [42]. Furthermore, self-report studies often experience high dropout rates, as respondents may find lengthy questionnaires difficult to complete [43].

Studies of this kind have considerable potential for confounding, and therefore, the conclusions drawn must be interpreted with caution. The modest sample and reliance on self-reported data all limit the strength of the inferences that can be made.

Hallucinations are frequently reported to intensify during periods of fatigue or heightened stress [30,32,33]. Findings from the present study suggest that physical activity may influence the perception and experience of stress, which in turn may contribute to a reduction in hallucinatory experiences. The relationship between physical activity, stress, and hallucinations is likely to reflect a complex interaction between neurophysiological processes and cognitive-perceptual mechanisms, indicating an important area for future investigation.

Chronic sleep deprivation has also been shown to induce hallucinations in severe cases [32,33]. This multifactorial issue warrants examination using more targeted and sensitive measures. The consequences of reduced sleep quality—including fatigue, impaired concentration, diminished physical stamina, and memory difficulties—represent additional variables that may contribute to hallucinatory experiences and therefore merit further exploration.

It is possible that physical activity levels are functioning as a proxy for other underlying factors, such as general health status, mobility, severity of visual impairment, or psychological wellbeing, that may more directly account for variation in hallucination severity. Further research is therefore required to disentangle these influences and to examine the contribution of additional variables, including mental health-related factors [27].

## 5. Conclusions

This study indicates that physical activity levels are low among individuals with CBS and suggests that physical activity may have the potential to influence the frequency of hallucinations experienced. Given the associations identified, further research involving a larger and more heterogeneous sample is planned to confirm these preliminary findings. To address the recruitment challenges encountered in the present study, more effective and targeted strategies for engaging individuals with CBS will be explored. Future work will also aim to determine the frequency, type, and intensity of physical activity required to produce meaningful reductions in hallucinatory experiences. In light of the low levels of physical activity reported, it will be important to examine barriers to physical activity in greater depth and to consider these as additional variables in subsequent investigations.

Overall, this study contributes to the growing body of literature emphasising the need for evidence-based management strategies for CBS, given the substantial distress that hallucinations can cause. The finding that higher levels of physical activity are associated with a reduced frequency or burden of hallucinations represents a meaningful addition to current understanding of CBS management and warrants further investigation. Moreover, the observation that physical activity was significantly associated with hallucination burden, whereas sleep disruption was not, merits deeper exploration.

## Figures and Tables

**Table 1:** Spearman’s correlations between hallucinations, physical activity, and sleep.

Variable Pair	Spearman’s Rho	*p* Value	Significance	Fisher’s z Effect Size	SE Effect Size
Hallucinations—PASE Activity	−0.335	0.011	*	−0.349	0.113
Hallucinations—Sleep	−0.018	0.894		−0.018	0.135
Hallucinations—Stress	0.417	0.001	*	0.445	0.139
PASE Activity—Sleep	−0.203	0.065		−0.206	0.137
PASE Activity—Stress	−0.497	<0.001	**	−0.545	0.140
Sleep—Stress	0.135	0.833		0.131	0.136

Note. All tests were two-tailed. * *p* < 0.01, ** *p* < 0.001.

## Data Availability

The data presented in this study are openly available in Figshare (10.6084/m9.figshare.32529780).

## Supplementary Materials

The following supporting information can be downloaded at: https://www.mdpi.com/article/10.3390/vision10030040/s1, Text S1: CBS questionnaire.
