# Supplementary material for: The Relationship Between Physical Activity, Sleep, and Hallucinations in Adults with Charles Bonnet Syndrome
Source: Vision (Basel). 2026 Jul 5;10(3):40. doi: 10.3390/vision10030040 (PMC13398291; doi:10.3390/vision10030040)
Supplement: Supplementary file 1 [file vision-10-00040-s001.zip › vision-4380459-supplementary.pdf]

The Relationship Between Physical Activity, Sleep, and Hallucinations in Adults with Charles Bonnet Syndrome

Jarrod Hollis [1], Rohit Narayan [1], Eldre W. Beukes [1], Rosie Lindsey [2], Aliyah Bharwani [1], Umair Mughal [1], Ikra Yaqoob [1], Justin A. Haegele [3], Scot Muirden [4] and Peter M. Allen [1,*]

CBS questionnaire

Start of Block: Personal Information

*

Q1. The next few questions are about personal information, such as ethnicity, age, and gender. Answer questions as best as you can. What is your age? (Type in the box below)

____

Q2. What is your gender? (Select the most appropriate)

- Male
- Female
- Non-binary
- Prefer not to say

Q3. What country do you reside in?

- UK
- USA

Q4. What is your race/ethnic group?

- White/ Caucasian
- Mixed or Multiple ethnic groups (Includes White and Black Caribbean, White and Black African, White and Asian, or any other Mixed or Multiple background)
- Asian or of Asian Decent (Includes Indian, Pakistani, Bangladeshi, Chinese, or any other Asian background)
- Black or of African or Caribbean descent
- Latino/a or other Hispanic ethnic groups
- Another racial/ethnic group (describe)

____

End of Block: Personal Information

Start of Block: About your vision

Q5. The next few questions are about your current vision status. Answer the questions to the best of your ability. Do you have a visual impairment?

- Yes
- No

Q6. Please specify the ocular condition(s) with which you have been diagnosed that have resulted in your visual impairment. (Select all that apply)

- ☐ Glaucoma
- ☐ Age-related macular degeneration
- ☐ Cataract
- ☐ Amblyopia
- ☐ Diabetic Retinopathy
- ☐ Retinitis Pigmentosa
- ☐ Other (please specify) ____

Q7. Are you considered legally blind in the country you reside in?

- ○ Yes
- ○ No

Q8. We had a patient here the other day who had a similar problem with their eyes to yours. The condition made it difficult to see things, and they noticed that they could see things that were really not there or that other people couldn't see (Throughout the survey, these will be known as visual phenomena). Has this ever happened to you?

- ○ Yes
- ○ No

Q9. Have you been told you have Charles Bonnet Syndrome by a health care professional?

- ○ No
- ○ Yes

Q10. Do you experience any visual phenomena? (Vivid, involuntary images appearing in your vision that you know are not real)

- ○ Yes
- ○ No

End of Block: About your vision

Start of Block: About your CBS

Q11. The next few questions are about your visual phenomena and their characteristics. Answer the next questions to the best of your ability. Do the visual phenomena impact the quality of your life/stop you from doing specific tasks?

- ○ Yes
- ○ No

Q12. What images were you seeing during a visual phenomenon? (Select all that apply)

- ☐ Complex Patterns
- ☐ Animals
- ☐ Plants, trees, foliage
- ☐ People
- ☐ Inanimate objects
- ☐ Coloured lights/ bright lights
- ☐ Palinopsia- seeing a ghost image
- ☐ Multiples of a real object
- ☐ Actual scenes from the past (e.g., your childhood)
- ☐ Insects/bugs
- ☐ Whole landscapes
- ☐ Faces/disembodied faces
- ☐ Distorted faces

Q13. How frequently do you experience visual phenomena?

- ◯ Daily
- ◯ Weekly
- ◯ Monthly
- ◯ Few episodes (specify) ______________________________

Q14. How long do the visual phenomena last?

- ◯ Seconds
- ◯ Minutes
- ◯ Hours
- ◯ Incessant or continuous

Q15. How long have you been experiencing visual phenomena?

- ◯ 1–3 months
- ◯ 4–6 months
- ◯ 6–12 months
- ◯ > 12 months

Q16. What are the lighting conditions during an episode of visual phenomena?

- [ ] Bright light
- [ ] Dim light
- [ ] Darkness
- [ ] Unsure

Q17. What are the circumstances at the onset of a visual phenomenon episode?

- [ ] Upon waking
- [ ] Relaxed state
- [ ] Alone at home
- [ ] Lifestyle changes
- [ ] Stress/excessive worry
- [ ] Unsure

Q18. What have you done to make the visual phenomena stop?

- ☐ Continue with activity
- ☐ Close eyes
- ☐ Blink
- ☐ Turn on lights
- ☐ Unsure

Q19. How stressful are the visual phenomena to you?

- ○ Mild
- ○ Moderate
- ○ Severe
- ○ Not stressful

Q20. Who have you told about these visual phenomena?

- ☐ Family member
- ☐ No one
- ☐ Ocular health professional
- ☐ Other (specify) ____

End of Block: About your CBS

Start of Block: Sleep in CBS

Q21. When thinking about how you sleep, for each of the following questions, please pick the answer that best describes you. Please rate your CURRENT (i.e., last 2 weeks) difficulty falling asleep.

- None (0)
- Mild (1)
- Moderate (2)
- Severe (3)
- Very severe (4)

Q22. Difficulty staying asleep?

- None (0)
- Mild (1)
- Moderate (2)
- Severe (3)
- Very severe (4)

Q23. Problems waking up too early?

- ◯ None (0)
- ◯ Mild (1)
- ◯ Moderate (2)
- ◯ Severe (3)
- ◯ Very severe (4)

Q24. How SATISFIED/DISSATISFIED are you with your CURRENT sleep pattern?

- ◯ Very satisfied (0)
- ◯ Satisfied (1)
- ◯ Moderately satisfied (2)
- ◯ Dissatisfied (3)
- ◯ Very dissatisfied (4)

Q25. How NOTICEABLE to others do you think your sleep problem is in terms of impairing the quality of your life?

- ◯ Not at all noticeable (0)
- ◯ A little (1)
- ◯ Somewhat (2)
- ◯ Much (3)
- ◯ Very much noticeable (4)

Q26. How WORRIED/DISTRESSED are you about your current sleep problem?

- ◯ Not at all worried (0)
- ◯ A little (1)
- ◯ Somewhat (2)
- ◯ Much (3)
- ◯ Very much worried (4)

Q27. To what extent do you consider your sleep problem to INTERFERE with your daily functioning (e.g., daytime fatigue, mood, ability to function at work/ daily chores, concentration, memory, etc.) CURRENTLY?

- ◯ Not interfering at all (0)
- ◯ A little (1)
- ◯ Somewhat (2)
- ◯ Much (3)
- ◯ Very much interfering (4)

Q28. When thinking about your visual phenomena and sleep, did you notice any changes in your visual phenomena if you sleep poorly? (Please select all that apply)

- ☐ Yes, INCREASED with good sleep
- ☐ Yes, DECREASED with good sleep
- ☐ Yes, INCREASED with poor sleep
- ☐ Yes, DECREASED with poor sleep
- ☐ No
- ☐ Not sure

End of Block: Sleep in CBS

Start of Block: Physical activity in CBS

Q29. The following questions are focused on the physical activities you do as part of your daily life. We appreciate that physical activity can vary according to the season; however, for this survey, we are only interested in the past 7 days. Do you think the amount of physical activity you do varies based on the season? If yes, please explain how and why this may vary in the box below.

- ○ Yes (explain) ____
- ○ No

Q30. What season are you currently in?

- Summer
- Winter
- Autumn/Fall
- Spring

Q31. Over the past 7 days, how often did you participate in sitting activities such as reading, watching TV, or doing handicrafts?

- Never
- Seldom (1–2 days)
- Sometimes (3–4 days)
- Often (5–7 days)

Page Break

Q32. What are these activities?

____

Q33. On average, how many hours per day did you engage in these sitting activities?

- ○ Less than 1 hour
- ○ 1–2 hours
- ○ 2–4 hours
- ○ More than 4 hours

Page Break

Q34. Over the past 7 days, how often did you take a walk outside your home or yard for any reason? For example, for fun or exercise, walking to work, walking the dog, etc.?

- ◯ Never
- ◯ Seldom (1–2 days)
- ◯ Sometimes (3–4 days)
- ◯ Often (5–7 days)

Page Break

Q35. On average, how many hours per day did you spend walking?

- ◯ Less than 1 hour
- ◯ 1–2 hours
- ◯ 2–4 hours
- ◯ More than 4 hours

Page Break

Q36. Over the past 7 days, how often did you engage in light sport or recreational activities such as bowling, golf with a cart, shuffleboard, fishing from a boat or pier, or other similar activities?

- Never
- Seldom (1–2 days)
- Sometimes (3–4 days)
- Often (5–7 days)

Page Break

Q37. What are these activities?

____

Q38. On average, how many hours per day did you engage in these light sports or recreational activities?

- ○ Less than 1 hour
- ○ 1–2 hours
- ○ 2–4 hours
- ○ More than 4 hours

Page Break

Q39. Over the past 7 days, how often did you engage in moderate sport and recreational activities such as double tennis, ballroom dancing, hunting, ice skating, golf without a cart, softball, or other similar activities?

- Never
- Seldom (1–2 days)
- Sometimes (3–4 days)
- Often (5–7 days)

Page Break

Q40. What are these activities?

____

Q41. On average, how many hours per day did you engage in these moderate sports and recreational activities?

◯ Less than 1 hour

◯ 1–2 hours

◯ 2–4 hours

◯ More than 4 hours

Page Break

Q42. Over the past 7 days, how often did you engage in strenuous sport and recreational activities such as jogging, swimming, cycling, singles tennis, aerobic dance, skiing (downhill or cross-country), or other similar activities?

- Never
- Seldom (1–2 days)
- Sometimes (3–4 days)
- Often (5–7 days)

Page Break

Q43. What were these activities?

____

Q44. On average, how many hours per day did you engage in these strenuous sports and recreational activities?

- ○ Less than 1 hour
- ○ 1–2 hours
- ○ 2–4 hours
- ○ More than 4 hours

Page Break

Q45. Over the past 7 days, how often did you do exercises specifically to increase muscle strength and endurance, such as lifting weights or pushups, etc.?

- Never
- Seldom (1–2 days)
- Sometimes (3–4 days)
- Often (5–7 days)

Page Break

Q46. What were these activities?

____

Q47. On average, how many hours per day did you engage in exercises to increase muscle strength and endurance?

- ○ Less than 1 hour
- ○ 1–2 hours
- ○ 2–4 hours
- ○ More than 4 hours

Q48. Are there any other conditions you may have that prevent you from doing these activities? (Apart from your visual impairment)

- ○ Yes (Describe) ____
- ○ No

End of Block: Physical activity in CBS

Start of Block: Household activities

Q49. The next few questions are about everyday exercises such as household chores and daily activities. During the past 7 days, have you done any light housework, such as dusting or washing dishes?

- ○ Yes
- ○ No

Q50. During the past 7 days, have you done any heavy housework or chores, such as vacuuming, scrubbing floors, washing windows, or carrying wood?

- ◯ Yes
- ◯ No

Page Break

Q51. During the past 7 days, did you engage in any of the following activities? Please answer YES or NO for each item. Home repairs, like painting, wallpapering, electrical work, etc.?

○ Yes (2)

○ No (1)

Q52. Lawn work or yard care, including snow or leaf removal, wood chopping, etc.?

○ Yes (2)

○ No (1)

Q53. Outdoor gardening?

○ Yes (2)

○ No (1)

Q54. Caring for another person, such as children, a dependent spouse, or another adult?

○ Yes (2)

○ No (1)

Page Break

Q55. During the past 7 days, did you work for pay or as a volunteer?

- ◯ No
- ◯ Yes

Page Break

Q56. How many hours per week did you work for pay and/or as a volunteer?

____

Q57. Which of the following categories best describes the amount of physical activity required on your job and/or volunteer work?

- ○ Mainly sitting with slight arm movements (Examples: office worker, watchmaker, seated assembly line worker, bus driver, etc.)
- ○ Sitting or standing with some walking (Examples: cashier, general office worker, light tool and machinery work)
- ○ Walking, with some handling of materials generally weighing less than 50 pounds (Examples: mailman, waiter/waitress, construction worker, heavy tool and machinery worker)
- ○ Walking and heavy manual work often requiring handling of material weighing over 50 pounds (Examples: lumberjack, stone mason, farm or general labourer)

Page Break

Q58. When thinking about your visual phenomena and physical activity, did you notice any changes in your visual phenomena after engaging in physical activity? (Please select all that apply)

- ☐ Yes, INCREASED with vigorous exercise
- ☐ Yes, DECREASED with vigorous exercise
- ☐ Yes, INCREASED with moderate exercise
- ☐ Yes, DECREASED with moderate exercise
- ☐ Yes, INCREASED with light exercise
- ☐ Yes, DECREASED with light exercise
- ☐ No
- ☐ Not sure
- ☐ Not applicable

End of Block: Household activities
